# The Bright Side of Gelatinous Blooms: Nutraceutical Value and Antioxidant Properties of Three Mediterranean Jellyfish (Scyphozoa)

**DOI:** 10.3390/md13084654

**Published:** 2015-07-29

**Authors:** Antonella Leone, Raffaella Marina Lecci, Miriana Durante, Federica Meli, Stefano Piraino

**Affiliations:** 1 Institute of Sciences of Food Production, National Research Council, Unit of Lecce (CNR, ISPA), Via Prov.le Lecce-Monteroni, 73100 Lecce, Italy; E-Mails: raffaella.lecci@ispa.cnr.it (R.M.L.); miriana.durante@ispa.cnr.it (M.D.); 2 Consorzio Nazionale Interuniversitario per le Scienze del Mare (CoNISMa), Local Unit of Lecce, Via Prov.le Lecce-Monteroni, 73100 Lecce, Italy; E-Mail: stefano.piraino@unisalento.it; 3 Dipartimento di Scienze degli Alimenti, Università di Parma, Parco Area delle Scienze, 59/A, 43124 Parma, Italy; E-Mail: federica.meli@campus-italy.com; 4 Università del Salento, DiSTeBA Via Prov.le Lecce-Monteroni, 73100 Lecce, Italy

**Keywords:** novel foods, marine jellyfish, macrozooplankton, antioxidants, collagen, nutraceuticals

## Abstract

Jellyfish are recorded with increasing frequency and magnitude in many coastal areas and several species display biological features comparable to the most popular Asiatic edible jellyfish. The biochemical and antioxidant properties of wild gelatinous biomasses, in terms of nutritional and nutraceutical values, are still largely unexplored. In this paper, three of the most abundant and commonly recorded jellyfish species (*Aurelia* sp.1, *Cotylorhiza tuberculata* and *Rhizostoma pulmo*) in the Mediterranean Sea were subject to investigation. A sequential enzymatic hydrolysis of jellyfish proteins was set up by pepsin and collagenase treatments of jellyfish samples after aqueous or hydroalcoholic protein extraction. The content and composition of proteins, amino acids, phenolics, and fatty acids of the three species were recorded and compared. Protein content (mainly represented by collagen) up to 40% of jellyfish dry weight were found in two of the three jellyfish species (*C. tuberculata* and *R. pulmo*), whereas the presence of *ω*-3 and *ω*-6 polyunsaturated fatty acids (PUFAs) was significantly higher in the zooxanthellate jellyfish *C. tuberculata* only. Remarkable antioxidant ability was also recorded from both proteinaceous and non proteinaceous extracts and the hydrolyzed protein fractions in all the three species. The abundance of collagen, peptides and other bioactive molecules make these Mediterranean gelatinous biomasses a largely untapped source of natural compounds of nutraceutical, cosmeceutical and pharmacological interest.

## 1. Introduction

In the last decades, the positive association between nutraceutical or functional food and human health prompted the search for bioactive compounds from living organisms. Although terrestrial plants and marine taxa represent the main sources of bioactive natural products, the inherent difficulties of sampling the diversity of aquatic environments have meant that the biochemistry of marine unicellular and multicellular organisms remains less explored [1,2,3]. The oceans represent the largest biome on earth, covering 71% of the planet surface, with a much higher diversity of body plans than on land [4]. Therefore, marine organisms still represent a largely unexplored reservoir of natural products, a vast potential source of diverse and healthy food, new drugs and bioactive compounds, due to the presence of secondary metabolites spanning a wide range of structural classes and various biosynthetic origins [5]. For this reason, many bioactive molecules from marine organisms with potential antimicrobial, anti-inflammatory and anticancer properties have represented the focus of recent researches [1,5,6,7,8,9]. 

Marine fishery resources have been exploited over a long time, leading to increasing vulnerability, irreversible change, and collapse of fish stocks [10,11]. Therefore, the search for new potential sources of bioactive compounds and their direct exploitation from living biomass primarily requires considerations on resource supply and its sustainable harvesting and must be supported by ecosystem-based studies of environmental sustainability to prevent overexploitation risks and ecological imbalances.

In this framework, cnidarians have increasingly become an important source of physiologically active compounds [8,9]. Particularly, jellyfish represent a conspicuous component of marine ecosystems and their populations are known to experience large seasonal and inter-annual fluctuations, characterized by sudden outbreaks—also known as “blooms”—followed by rarity periods [12]. In recent years, evidence is accumulating concerning increasing magnitude and frequency of jellyfish blooms [13,14], which represent a widely distributed and abundant gelatinous biomass in the world oceans. Jellyfish blooms are mainly sustained from large Rhizostomoidea and Semaeostomeae medusae at all latitudes of the Southern and Northern hemispheres, with many coastal locations sustaining high gelatinous biomasses directly influenced by global warming [15,16,17]. From an anthropocentric point of view, jellyfish outbreaks are often negatively perceived because they (i) may determine severe negative consequences on human health and coastal tourism due to painful stings; (ii) may impair coastal industries by clogging cooling pipelines [18]; (iii) affect fishery by the reduction of fish stocks through competition for food or direct predation [19,20]; or (iv) produce mass mortalities in caged fish aquaculture. Management and adaptation strategies have been developed aiming to prevent negative impacts [17,21] also in the framework of dedicated research projects, like the MED-JELLYRISK project within ENPI CBC MED (European Neighbourhood and Partnership Instrument Cross-Border Cooperation in the Mediterranean) [22]. However, due to their high abundances and high regenerative and reproductive potentials, jellyfish may be regarded as a new source of pharmacologic, nutraceutical and food/feed compounds, and their potential use in tissue engineering, food industry and medicine may provide the opportunity of showcasing jellyfish in a more positive light [12,23,24,25,26]. Information on the biochemical composition and biomass of jellyfish has been available in recent years. The dry mass (DW) is in the range of about 3%–5% of the fresh weight, and jellyfish carbon (C) is typically <15% of DW [27] where in non-gelatinous groups it accounts for up to 30%–60% [28]. The organic content is mainly represented by protein (collagen) while lipids and carbohydrates represent minor components of jellyfish tissue [29,30].

Despite a low organic content, jellyfish have long been highly considered in Asiatic countries for their therapeutic value in the treatment of arthritis, hypertension, bone pain, and ulcers, as softening skin and improving digestion [31,32]. These properties, described mainly in non-scientific publications [33], are likely attributable to the collagen, a structural protein family widely present throughout the animal tissues as prevailing component of extracellular matrices in connective tissues [34,35] and the main structural protein in the jellyfish body mass [32,36]. Collagen has diverse general and biomedical applications and is also a common constituent of many cosmetic and food products in the form of gelatin. Interest in collagen as a biomaterial is due to its low immunogenicity and high biocompatibility [37], and because it can be extracted from a variety of organisms, such as bovine and porcine skins. However, these sources of collagen are increasingly rejected for disease risks (like bovine spongiform encephalopathy) or religious reasons, while marine organisms, especially marine invertebrates are becoming an attractive source for collagen industrial uses. Structure and sequences of fibrillar collagen are highly conserved, and cnidarian collagen shares several features with their human counterparts [38]. Jellyfish collagen from *Rhopilema esculentum* could protect mice skin from the ultra-violet (UV) radiation damages alleviating the UV-inducing abnormal changes of antioxidative indicators [39,40]. Collagen from the giant edible jellyfish *Nemopilema nomurai* showed immunostimulatory effect *in vitro*, on hybridoma line HB4C5, human peripheral blood lymphocytes [41], and *in vivo* [42]. In addition, the oral administration of type II-like collagen of cannonball jellyfish *Stomolophus meleagris* delayed the onset and suppressed collagen-induced arthritis in animal models [43]. More recent studies have shown medical properties of this polymer extracted from *Rhopilema esculentum* for cartilage tissue engineering [44]. Accordingly, jellyfish collagen might be also used in the cosmetics, in creams and lotions for the skin as well as in the biomedical and pharmaceutical industry.

Collagen can also be a source of bioactive peptides. Bioactive peptides are 2–20 amino acid fragments inactive in the parent protein; when released by enzymatic hydrolysis, these fragments may exert various physiological functions, depending on their specific amino acid composition [45]. Food derived peptides may have several functions such as immunomodulatory [46], antimicrobial [47], antioxidative [48], and antihypertensive [49] properties. Due to their high bioactivity and biocompatibility, collagen peptides and hydrolysate may be used as functional ingredients in medicine and food industries. Collagen hydrolysate of the jellyfish *Rhopilema esculentum* has shown antioxidant activity, ability to chelate Cu^2+^ ions and to inhibit tyrosinase activity [39]. Enzymatic hydrolysis, indeed, may improve the functional properties of proteins, such as solubility and emulsification [50]. Enzymatic hydrolysates from jellyfish collagen are known to have protective effects on mice skin photoaging induced by UV irradiation higher than non-hydrolyzed jellyfish collagen [40]. This mechanism is probably related to the *in vivo* antioxidative properties showed by collagen and peptides with high contents of glycine, proline, and hydrophobic amino acids [51].

Besides specific properties of the proteins and derived peptides, the whole mass of jellyfish, as other marine products, might be considered for food or feed purpose due to their content of essential nutrients or biochemical characteristics unavailable or poorly present in products from terrestrial plants and animals. Indeed, several species of the scyphozoan jellyfish with middle stings in South-East Asia, mainly in China and in Japan, represent a part of the multimillion-dollar seafood business and are appreciated not only for its texture and taste, but also for its composition which ensures a low calorie diet being low in fat, cholesterol, and salt [31,32,33]. 

Seafood deserves a key role in nutrition and health because it provides omega-3 and omega-6 fatty acids known for the reduction power of cholesterol levels and the decrease of incidence of coronary heart diseases. The lipid composition of several cnidarians may vary substantially [52,53], depending on diet or symbiotic association with unicellular algae. For instance, lipid composition seems determined by diet in non-symbiotic jellyfish, *i.e.*, the moon jellyfish *Aurelia* sp*.* [54]. Conversely, in zooxanthellate cnidarians lipids are regularly translocated in their tissues from their unicellular symbionts [8,55]. The absence of storage lipids, such as wax esters, also suggests that proteins govern energy storage [56,57]. Remarkably, jellyfish feed used for chickens and pigs determined an increase of muscle to bone ratio and of the overall body tissue without toxic effect on blood, liver and muscle [32].

In this study, original data on the biochemical composition and nutraceutical properties of three jellyfish, namely the scyphozoan *Aurelia* sp.1 (commonly known as moon jellyfish), *Cotylorhiza tuberculata* (known as fried-egg jellyfish) and *Rhizostoma pulmo* (known as sea lung jellyfish) are provided. These species bloom yearly along the Mediterranean coastal areas from Spain to the North Adriatic Sea, forming large populations of considerable and totally unexploited biomass, thus representing excellent candidates for the isolation and potentially sustainable production of bioactive compounds in the fields of nutraceuticals, animal feeds, and pharmaceutics. Quali-quantitative identification and measurement of proteins, together with their antioxidant activity, and analysis of lipid content were carried out to assess biochemical values of these gelatinous organisms as putative novel food or for the production of jelly-related, low-cost raw materials for either animal feed or for applications in cosmetics or biomedical industries. 

## 2. Results and Discussion

### 2.1. Jellyfish Blooms and Biomass Characterization

The semaeostome jellyfish *Aurelia* sp.1 can be found in marinas and coastal lagoons of the Mediterranean Sea. This is a non-indigenous or alien species introduced in the Mediterranean Sea by shellfish aquaculture, by the transfer of the polyp stage commonly living on bivalve shells. In the Varano lagoon (Apulia, SE Italy), a dense population of *Aurelia* sp.1 medusae (up to 80 individuals·m^−3^) is generated yearly from February to July. Differently, the rhizostome jellyfish *Cotylorhiza tuberculata* and *Rhizostoma pulmo* [58] are typical species of marine coastal waters and can be encountered across the Western and Central Mediterranean Sea. These two species are among the top five most frequently recorded species along the Italian coastlines by the citizen science project METEOMEDUSE carried out by the MED-JELLYRISK project [22] and represent the largest part of jellyfish biomass off the Apulia coasts across the years 2010–2014. In September 2013, a high density (>48,000 individuals/km^2^) of *R. pulmo* jellyfish was assessed along the Apulian shores in southwestern part the Gulf of Taranto by an ultralight aerial survey in the framework of the MED-JELLYRISK project [22], with an estimated biomass range of 100–300/km^2^ (MED-JELLYRISK, unpublished data). Biometric and average biomass data of individuals of the three jellyfish species are shown in Table 1.

**Table 1:** Biometric measures, fresh and dry weights and organic matter of the three jellyfish species sampled in the 2010–2014 summers.

Jellyfish Samples	Umbrella Diameter Range *	Fresh Weight Range *	Ratio FW/Diameter	Range of DW *	Organic Matter (OM) Mean **
	Mean (cm)	Mean (g)		(% of FW)	(% of DW)
*Aurelia* sp.1	10–23 *16.2 ± 4.9*	47–604 *257 ± 237*	6.8–26.3 13.5 ± 9.2	2.2–3.0	23.9 ± 3.3 ^a^
*Cotylorhiza tuberculata*	6–29 *17.7 ± 6.3*	19–1770 *638 ± 475*	3–61 24.3 ± 16.9	3.9–32.4	30.2 ± 2.4 ^b^
*Rhizostoma pulmo*	8–37 *20.8 ± 7.2*	42–2440 *860 ± 720*	5.3–65.9 32.6 ± 14.2	4.1–6.8	29.5 ± 6.6 ^b^

FW, fresh weight; DW, dry weight; * Data are expressed as range and/or means ± standard deviation (10 < *n* < 41); ** Organic matter data are the mean of two independent experiments each performed in quintuplicate, superscript lower case letters indicate significant differences (*p* < 0.05) within the column.

The umbrella diameter of *Aurelia* sp.1 specimens ranged from about 10–23 cm, *C. tuberculata* and *R. pulmo* umbrellas were 6–29 cm and 8–37 cm, respectively, with a proportionally increasing biomass with jellyfish size. The variability of the biometric measures, including the fresh-weight (FW)/diameter ratio and dry weight (DW) percentage values, is representative of the seasonal growth of jellyfish collected at different times throughout the spring-summer months and at different growth stages [8]. After lyophilisation, the DW of *Aurelia* sp.1 from Varano ranged from 2.2% to 3%, *C. tuberculata* 3.9%–32.4% and *R. pulmo* 4.1%–6.8% of the FW, displaying a high and quite constant water content for *Aurelia* sp.1 and *R. pulmo* and high variability in tissue consistency in *C. tuberculata* specimens, which reached also the highest DW proportion. The DW of *Aurelia* sp.1 from Varano was slightly lower than for moon jellyfish collected along the Slovenian coasts (4.0% of FW) whereas similar results were obtained for *R. pulmo* [59]*.* The organic matter content (OM) was highly comparable between *C. tuberculata* and *R. pulmo* (30.2% ± 2.4% and 29.5% ± 6.6% of DW, respectively), while *Aurelia* sp.1 (23.9% ± 3.3% of DW) showed a statistically significant lower OM than *C. tuberculata*. The fried-egg jellyfish also showed higher DW values than *R. pulmo* or *Aurelia* sp.1, providing critical information for the potential exploitation of these jellyfish biomass.

The potential use of jellyfish biomass should be assessed also by taking into consideration the total energy or gross energy value (GE), which is directly related to DW and OM values. The GE of jellyfish biomass has long been neglected and poorly documented, compared to other planktonic taxa with prominent roles in marine food webs, such as crustaceans or fish, and only a few studies dealt with jellyfish GE to assess their value as a prey for apical gelativorous predators, such as fish or turtles. This is because the energetic value of jellyfish biomass was long considered poor compared to other prey items. However, recent evidence shows jellyfish biomass may represent a key component of diet of several organisms: leatherback sea turtles may consume up to 261 jellyfish·day^−1^ (330 kg jellyfish wet mass·day^−1^), whereas several fish species may rely on jellyfish prey up to 100% of their diet [60,61,62]. Measured by bomb calorimetric or calculated from carbon content, the GE for *Cyanea capillata*, *Rhizostoma octopus* and *Chrysaora hysoscella* [63], *Atolla wyvillei*, *Aurelia aurita* and *Pelagia noctiluca* [64], was in the range of 2.3–5.95 kJ/g of DW, with differences between species and body parts. These values are lower than the range of energy values of protein (10.2–18.2 kJ/g), fat (35.0–37.7 kJ/g) and total carbohydrate (11.3–17.4 kJ/g) in ordinary human diet [65], and actually lower than other marine organisms used as human foods or animal feed. In this framework, jellyfish may represent a healthy energy-restricted food that may reduce caloric intake and over-nutrition trends in the typical Western human lifestyle, without necessarily decreasing the amount of consumed food [66].

### 2.2. Jellyfish Protein

#### 2.2.1. Amino Acid Composition

The amino acid (AA) composition has been determined for the three species of jellyfish (Table 2). None of the jellyfish protein samples contained the essential amino acid (EAA) tryptophan (Try), as previously reported for collagen peptides derived from *Rhopilema esculentum* umbrella [67] and gonads [68], whole tentacles and nematocyst suspensions of *Chrysaora quinquecirrha* [69], and total proteins profiles from *Chrysaora hysoscella*, *Pelagia noctiluca* and also *R. pulmo* [59]*.* All the remaining EAA, namely histidine (His), isoleucine (Ile), leucine (Leu), lysine (Lys), methionine (Met), phenylalanine (Phe), threonine (Thr) and valine (Val), were found in *R. pulmo* and *C. tuberculata* specimens. Differently, the EAAs His and Leu were not detected in *Aurelia* sp.1, as well as cysteine (Cys) and arginine (Arg) were not found in *C. tuberculata* (Table 2).

The proportion of EAAs out of the total AAs in the whole tissues of the *Aurelia* sp.1, *C. tuberculata* and *R. pulmo*, was 31.4%, 53.6%, and 50.8%, respectively. The last two EAA percentages were higher than those recorded from the gonads of the edible Asiatic jellyfish *Rhopilema esculentum* [68], which may depend on the advanced reproductive status of many jellyfish specimens sampled during this study. Overall, the EEAs content of the three Mediterranean jellyfish is comparable to those recorded in other high-value Asiatic and European seafood, at least in terms of percentage composition [68,70].

The most abundant amino acid found in *Aurelia* sp.1 was Gly, followed by Glu, Ser, Thr and Tyr (Table 2). Gly is the fixed constituent of collagen-typical repeating triplets with a repeating X-Y-Gly sequence, where X and Y can be any amino acid, although proline (Pro) and hydroxyproline (Hyp) residues are the most common triplet in collagen [71,72]. The amounts of aromatic amino acids (AAA) were 12.7%, 22.9% and 22.5% of the total amino acids in *Aurelia* sp.1, *C. tuberculata* and *R. pulmo*, respectively. The AA profiles were more similar between the two rhizostome jellyfish *C. tuberculata* and *R. pulmo*, both as EAA and AAA percentages, as well as single AAs, where glutamine/glutamate were the most representative followed by Phe, Leu, Tyr, Thr, His and Ser (Table 2). The putative occurrence of a wider diversity of proteins other than collagen in the two Rhizostomeae could justify their different AA profiles compared to *Aurelia* sp.1. Overall, the finding of important proteinogenic and non-proteinogenic AAs (such as Glu, Gly, Phe, Asp, Met, Leu, Tyr, Lys, and Arg) may account for the long tradition of therapeutic value of jellyfish food in Chinese pharmacopeia.

**Table 2:** Amino acid profiles of the total proteins in jellyfish samples of *Aurelia* sp.1, *Cotilorhiza tuberculata* and *Rhizostoma pulmo*. Data are expressed as mean of three replicates as mg/100 g of dry powder ± standard deviation (SD) and as percentage of total amino acids.

	*Aurelia* sp.1		*C. tuberculata*		*R. pulmo*	
	mg/100 g ± SD	%	mg/100 g ± SD	%	mg/100 g ± SD	%
Alanine (Ala)	7.1 ± 0.3	**4.5**	2.2 ± 0.2	**4.3**	3.5 ± 0.2	**3.9**
Arginine (Arg)	1.1 ± 0.0	**0.7**	n.d.	**-**	1.8 ± 0.0	**2.0**
Aspartic acid + Asparagine (Asx) *	3.2 ± 0.2	**2.0**	1.3 ± 0.1	**2.5**	2.9 ± 0.6	**3.2**
Cysteine (Cys)	4.1 ± 0.2	**2.6**	n.d.	**-**	1.2 ± 0.0	**1.3**
Glutamic acid + Glutamine (Glx) **	13.6 ± 0.3	**8.7**	8.2 ± 0.6	**16.0**	13.7 ± 0.2	**15.2**
Glycine (Gly)	55.4 ± 1.1	**35.2**	3.0 ± 0.1	**5.9**	4.8 ± 0.5	**5.3**
Histidine (His) ^e^	n.d.	**-**	4.0 ± 0.1	**7.8**	5.0 ± 0.4	**5.6**
Isoleucine (Ile) ^e^	6.8 ± 0.3	**4.3**	2.9 ± 0.5	**5.7**	4.9 ± 0.7	**5.5**
Leucine (Leu) ^e^	n.d.	**-**	3.8 ± 0.6	**7.4**	8.2 ± 0.4	**9.1**
Lysine (Lys) ^e^	9.4 ± 0.3	**6.0**	3.1 ± 0.5	**6.1**	6.2 ± 0.4	**6.9**
Methionine (Met) ^e^	5.9 ± 0.9	**3.8**	2.7 ± 0.5	**5.3**	4.1 ± 0.7	**4.6**
Phenylalanine (Phe) ^e^	10.4 ± 0.3	**6.6**	4.1 ± 0.2	**8.0**	8.4 ± 0.8	**9.3**
Proline (Pro)	4.3 ± 0.3	**2.7**	2.6 ± 0.3	**5.1**	3.5 ± 0.2	**3.9**
Serine (Ser)	9.5 ± 0.4	**6.0**	2.8 ± 0.0	**5.5**	6.0 ± 0.8	**6.7**
Threonine (Thr) ^e^	10.1 ± 0.9	**6.4**	3.8 ± 0.0	**7.4**	4.5 ± 0.1	**5.0**
Tyrosine (Tyr)	9.5 ± 0.2	**6.0**	3.6 ± 0.2	**7.0**	6.8 ± 0.6	**7.6**
Tryptophan (Try) ^e^	n.d.	**-**	n.d.	**-**	n.d.	**-**
Valine (Val) ^e^	6.8 ± 0.5	**4.3**	3.0 ± 0.3	**5.9**	4.4 ± 0.4	**4.9**
∑_AA_	157.2 ± 6.2	**100**	51.1 ± 4.2	**100**	89.9 ± 7.0	**100**
∑_EAA_	49.4 ± 3.2	**31.4**	27.4 ± 2.7	**53.6**	45.7 ± 3.9	**50.8**
∑_CAA_	93.4 ± 2.3	**59.4**	20.2 ± 1.2	**39.5**	36.6 ± 2.3	**40.7**
∑_AAA_	19.9 ± 0.5	**12.7**	11.7 ± 0.5	**22.9**	20.2 ± 1.8	**22.5**

* As sum of aspartic acid and asparagine; ** As sum of glutamic acid and glutamine; ^e^ essential amino acids; ∑_AA_, total amino acids; ∑_EAA_, sum of essential amino acids; ∑_CAA_, sum of conditionally essential amino acids; ∑_AAA_, sum of aromatic amino acids; SD, standard deviation; n.d., not detected.

#### 2.2.2. Protein Content and Composition

The total freeze-dried tissue was subjected to different solvent extractions and sequential proteolytic digestion to compare the protein content and composition of the three jellyfish species. The whole tissues of *Aurelia* sp.1, *C. tuberculata* and *R. pulmo* contained about 57, 22, and 60 mg of proteins/g of DW, respectively (Table 3). All the three jellyfish species contained proteins soluble in polar solvents, especially proteins soluble in aqueous solution (phosphate buffered saline, PBS) (32%–60% of total proteins) significantly more abundant than proteins soluble in 80% ethanol or methanol (3.4%–10.5% of the total proteins). The fried-egg jellyfish *C. tuberculata* showed the highest percentage of both PBS- and hydroalcoholic-soluble proteins, as compared to *Aurelia* sp.1 and *R. pulmo*.

**Table 3:** Composition of biomasses from the three jellyfish species *Aurelia* sp.1, *C. tuberculata* and *R. pulmo*. The polar solvent extraction with 80% methanol (treatment A) or 80% ethanol (treatment B) or phosphate buffered saline (PBS) (treatment C) was followed by enzymatic digestion with pepsin and then collagenase. Data are presented as the mean of three independent experiments in triplicate and are expressed as mg of protein per gram of dry weight ± standard deviation (SD) and as percentage of the total proteins. Chicken collagen was used for comparison and as a control for the hydrolysis reactions.

		*Aurelia* sp.1	*Cotylorhiza tuberculata*	*Rhizostoma pulmo*	Collagen (Chicken Cartilage) (*%*)
	Treatments	mg of proteins/g of dry weight (*% of total proteins*)			
Soluble proteins	A—80% MeOH B—80% EtOH C—PBS	3.8 ± 0.8 (*6.9)* 3.9 ± 0.4 (*7.7*) 22.3 ± 1.1 (*32.2*)	2.4 ± 0.5 (*8.3*) 2.8 ± 0.8 (*10.5*) 35.4 ± 4.6 (*59.1*)	2.0 ± 0.9 (*3.4*) 2.1± 1.0 (*3.4*) 37.4 ± 6.6 (*38.5*)	- - -
Pepsin digestible proteins	Treatment A Treatment B Treatment C Non Treated	12.2 ± 2.9 (*22.1*) 10.4 ± 0.9 (*20.6*) 7.3 ± 0.2 (*10.6*) 14.5 ± 2.0 (*25.5*)	6.4 ± 3.2 (*22.2*) 6.7 ± 3.2 (*25.1*) 4.5 ± 1.4 (*7.6*) 4.3 ± 0.9 (*19.4*)	18.6 ± 3.0 (*32.4*) 20.6 ± 3.5 (*33.4*) 19.9 ± 4.4 (*20.5*) 19.6 ± 4.7 (*32.5*)	(*0.7*)
Collagenase hydrolysable proteins	Treatment A Treatment B Treatment C Non Treated	38.0 ± 0.6 (*69.1*) 35.3 ± 0.9 (*69.9*) 38.8 ± 1.8 (*55.8*) 40.5 ± 0.5 (*71.0*)	18.4 ± 0.8 (*63.1*) 14.6 ± 0.8 (*55.0*) 18.3 ± 0.7 (*30.6*) 15.7 ± 4.4 (*70.8*)	33.6 ± 5.1 (*58.6*) 35.6 ± 4.3 (*57.6*) 35.2 ± 6.9 (*36.2*) 36.6 ± 7.0 (*60.8*)	(*99.3*)
Not-hydrolyzed proteins	Treatment A Treatment B Treatment C Non Treated	1.1 ± 0.1 (*1.9*) 1.9 ± 0.1 (*1.8*) 1.0 ± 0.1 (*1.5*) 2.0 ± 0.2 (*3.6*)	1.9 ± 0.3 (*6.4*) 2.5 ± 0.4 (*9.5*) 1.6 ± 0.1 (*2.8*) 2.2 ± 0.3 (*9.8*)	3.2 ± 0.3 (*5.6*) 3.5 ± 0.9 (*5.6*) 4.7 ± 1.3 (*4.9*) 4.0 ± 0.9 (*6.7*)	-
Total	Treatment A Treatment B Treatment C Non Treated	55.1 (*100*) 50.5 (*100*) 69.5 (*100*) 57.0 (*100*)	29.1 (*100*) 26.6 (*100*) 59.9 (*100*) 22.2 (*100*)	57.5 (*100*) 61.8 (*100*) 97.2 (*100*) 60.1 (*100*)	(*100*)

MeOH, methanol; EtOH, ethanol; PBS, phosphate buffered saline.

**Scheme 1:**
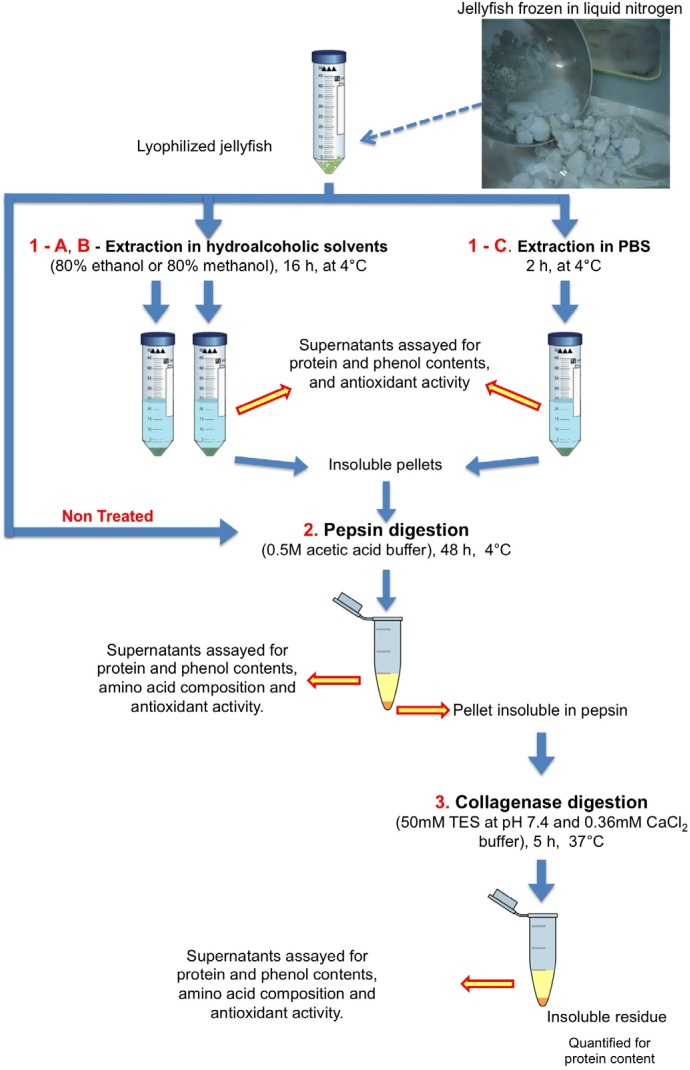
Protocol set up for the lyophilized jellyfish “sample extraction” by hydroalcoholic solvents or phosphate buffered saline (PBS), followed by sequential enzymatic hydrolysis of proteins.

To estimate the potential of these jellyfish species as a source of high value and digestible peptides, a sequential protein extraction procedure, by means of consecutive enzymatic hydrolysis, was designed. A digestion with pepsin was performed as first step on jellyfish samples pre-extracted by aqueous or hydroalcoholic solutions, or on non-treated jellyfish tissues, followed by the collagenase digestion (Scheme 1). The *in vitro* pepsin hydrolysis roughly mimics the gastric phase of the human digestion, establishing the rate of proteins readily digestible in the jellyfish biomass. The soluble pepsin-digested peptides were recovered in the supernatant, while undigested and insoluble proteins remained in the precipitate and were subjected to collagenase digestion. Collagen from chicken cartilage was subjected to the same procedure as a control for the hydrolysis reaction and for comparison with a common food collagen protein. Ranges of 20%–33% of the jellyfish total proteins were pepsin-digestible peptides, while the remainder consisted mainly of collagen (60%–71% of the total proteins) (Table 3). Only a small amount of proteins was detected as non-digested residue (about 2%–9% of the total proteins), most of them occurring in the untreated samples (without pre-extraction), putatively indicating a limited accessibility to the enzymes, rather than indigestibility. These results suggest that a significant proportion of both collagen- and pepsin-digestible jellyfish peptides can be dispersed in aqueous solutions by PBS pre-extraction, while the pre-treatments with methanol and ethanol solutions allow limited extraction of proteins. Indeed, treatment with ethanol-based solutions can represent a method to remove fats and pigments [8], leaving the protein fraction, including collagen, quite clean. Indeed, partially fractionated hydroalcoholic extracts from *C. tuberculata* jellyfish contained carotenoids and fatty acids derived by algal symbionts, and yielded compounds with biological activity, representing a high potential protocol for nutraceuticals and drug discovery [8].

The most abundant peptides in all three jellyfish samples are obtained from collagen digestion (Table 3). Jellyfish collagens were generally classified according to homology with vertebrate type I, II, or type V, depending on jellyfish species and collagen classification, and have been recognized as very suitable material for cartilage tissue engineering and other biomedical applications [36,44,73,74,75,76,77,78,79].

The pre-digestion with pepsin and the evaluation of only collagenase-digested polypeptides implies that just pure collagen was evaluated. It is noteworthy that *Aurelia* sp.1 and *R. pulmo* tissues contained up to about 40% of pure collagen based on the lyophilized dry weight. That percentage is consistent with previous reported data on different species of edible Asiatic jellyfish, obtained after pepsin digestion [74,78,80] with a yield of collagen of 46.4% based on the lyophilized dry weight for *Stomolophus meleagris* [74] and 35.2% for *Rhopilema asamushi* [80]. A pepsin-solubilized collagen was obtained from *Chrysaora* sp. with a maximum yield of 19% (ash-free lyophilized dry weight) [74]. Also, the pre-digestion by pepsin leads to the elimination of non-helical terminal regions of collagen (telopeptides), which are also the major antigenic determinants giving a collagen product (atelocollagen) with high purity and increased solubility. The reduced degree of antigenicity and the solubility features of the jellyfish-derived collagen have also prompted the use of collagen in the food and cosmetics sector [1,72,77].

Enzymatic hydrolysis of seafood and other fish biomass has been employed as an alternative approach to the conversion of underutilized biomass or by-products into edible protein products [81], and similar processing methodologies might be used to successfully exploit the exceedingly large amount of jellyfish biomasses.

### 2.3. Phenolic Compound Content in Jellyfish Hydroalcoholic Soluble Extracts

**Figure 1:**
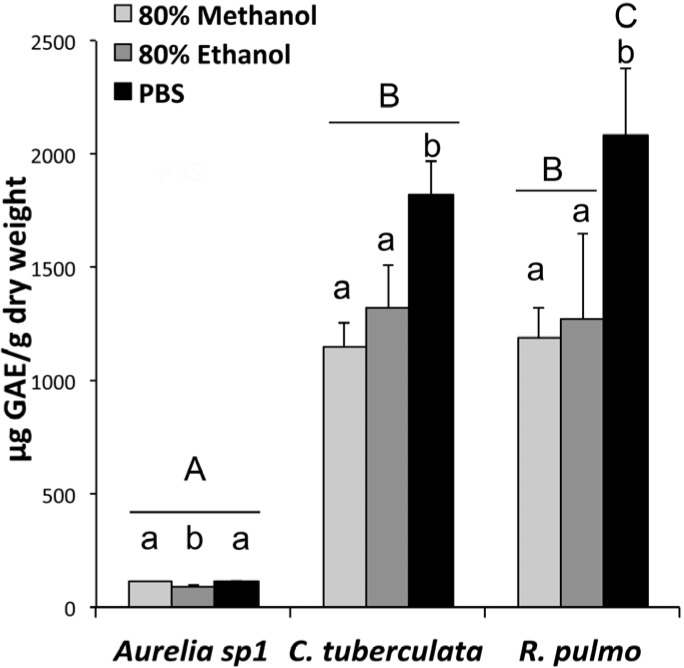
Total phenolic compounds in jellyfish extracted with phosphate buffered saline (PBS), 80% methanol and 80% ethanol from freeze-dried tissues of *Aurelia* sp.1, *C. tuberculata* and *R. pulmo*. Data are expressed as μg of gallic acid equivalents (GAE) per gram of dry weight and are means of three independent experiments performed in triplicate, bars represent mean ± standard deviation (SD). A,B,C: the different capital letters indicate differences among species for the same extraction type; a,b,c: the different lower case letters indicate significant differences among extracts in the same jellyfish species, (*p* < 0.05).

The total phenolic content of the jellyfish extracts was significantly different among the three jellyfish species (Figure 1). In all extracts from *Aurelia* sp.1 samples, a low content of total phenols was detected, as compared to the other two jellyfish species, with 113.2 ± 0.4 μg GAE (gallic acid equivalent) per gram of DW in the 80% methanol extract, 86.4 ± 9.4 μg GAE/g in the 80% ethanol extract and 115.5 ± 2.0 μg GAE/g in the PBS extract. The total phenol content detected in all extracts of both *C. tuberculata* and *R. pulmo* was significantly higher than in *Aurelia* (Figure 1)*.* The highest concentration was detected for both species in the PBS extracts, reaching 1817.7 ± 153.7 μg GAE/g DW and 2079.3 ± 301.9 μg GAE/g DW, for *C. tuberculata* and *R. pulmo*, respectively.

The presence of phenolic compounds in jellyfish is yet poorly documented. Phenols were detected in the podocyst cuticle of *Chrysaora quinquecirrha* [82], and benzene-1,2-dicarboxylic acid or phthalates were detected in the adult tissues of *Cyanea capillata* and *Chrysaora quinquecirrha* [83] as well as in *C. tuberculata* extracts [8]. Recent data showed that polyphenols may enhance the biostability and biomechanical properties of collagen based tissues by modulation of mechanisms of collagen fibers cross-linking at molecular, inter-molecular and inter-microfibrillar levels [84,85]. There is a remarkable difference in the stiffness and consistency of jellyfish extracellular matrix between the highly flexible and soft *Aurelia* spp. jellyfish against the robust and hardened mesoglea of *C. tuberculata* and *R. pulmo*, and it can be hypothesized that the higher observed concentration of phenols in the large rhizostomate jellies may be responsible for or contribute to the evolution of different jellyfish functional and anatomical adaptations.

The high phenolic content in the PBS extracts could also be related to the measurements of phenolic amino acidic residues of the proteins. However, the observed differences of total phenol contents between the three jellyfish species is not paralleled by differences in protein contents, suggesting that the two rhizostome species really contain higher phenol concentrations than *Aurelia* sp.1.

### 2.4. Antioxidant Activity

**Figure 2:**
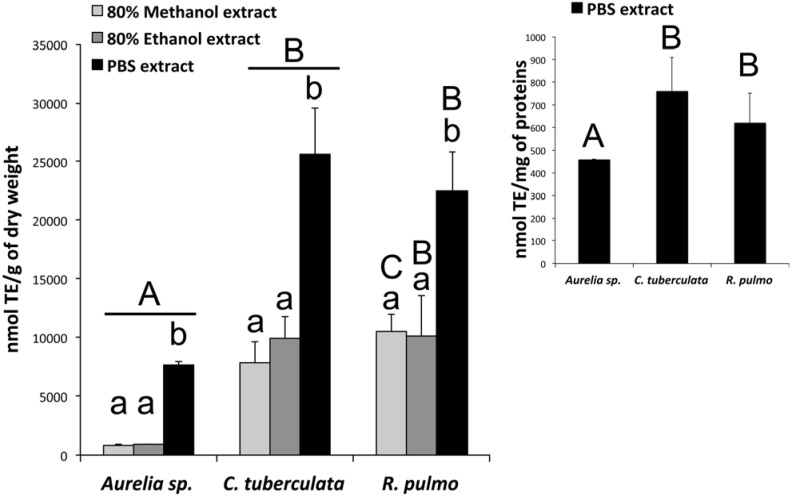
Total antioxidant activity in jellyfish extracted with phosphate buffered saline (PBS), 80% methanol and 80% ethanol from freeze-dried tissues of *Aurelia* sp.1, *C.* *tuberculata* and *R. pulmo*. Antioxidant activity is expressed as nmol of Trolox eq. (TE) per gram of dry weight or as nmol TE per mg of proteins (inset). Data are the mean of three independent experiments performed in triplicate, bars represent mean ± standard deviation. A,B,C: different capital letters indicate differences among species for the same extraction type; a,b,c: the different lower case letters indicate significant differences among extracts in the same jellyfish species, (*p* < 0.05).

**Figure 3:**
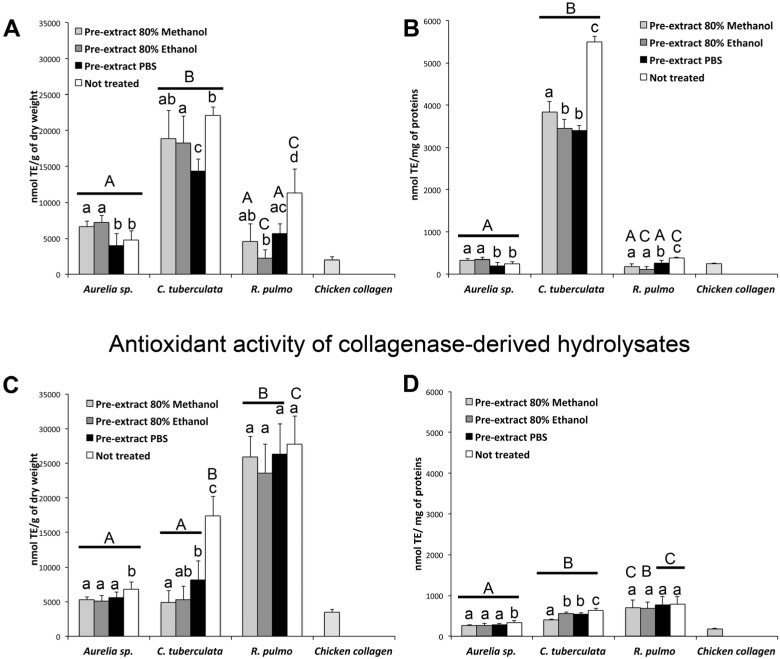
Total antioxidant activity in pepsin hydrolyzed proteins (**A**,**B**) and collagenase hydrolyzed peptides (**C**,**D**) from freeze-dried tissues of *Aurelia* sp.1, *C. tuberculata* and *R. pulmo*. Chicken collagen was hydrolyzed in parallel and used as a comparison. Antioxidant activity is expressed as nmol of Trolox eq. (TE) per gram of dry weight (DW) (**A**,**C**) or as nmol TE per mg of proteins (**B**,**D**). Data are the mean of three independent experiments performed in triplicate, bars represent mean ± standard deviation (SD). A,B,C: the different capital letters inside the figures indicate differences among species for the same treatment type; a,b,c: the different lower case letters inside the figures indicate significant differences among treatments in the same jellyfish species, (*p* < 0.05).

A significant antioxidant activity, measured as radical scavenging activity, was detected in all samples including the jellyfish aqueous/hydroalcoholic extracts and the hydrolyzed peptides resulting from pepsin and collagenase digestions (Figure 2 and Figure 3).

#### 2.4.1. Antioxidant Activity in Aqueous and Hydroalcoholic Extracts

The total antioxidant activity in the PBS and hydroalcoholic extracts (Figure 2) was much higher in *C. tuberculata* and *R. pulmo* than in *Aurelia* sp.1, mostly detected in the PBS extracts. In *Aurelia* samples, the antioxidant activity ranged around 833 ± 2.31 nmol of TE (Trolox Equivalent)/g DW in the methanol extract, 890 ± 3 nmol of TE/g DW in the ethanol extract and 7651 ± 349 nmol of TE/g DW in the PBS extract. In methanol, ethanol and PBS extracts of *C. tuberculata* the antioxidant activity was 7895 ± 1778 nmol TE/g DW, 9933 ± 1898 nmol TE/g DW and 25,621 ± 3959 nmol of TE/g DW, respectively. In *R. pulmo* ranged from about 10,000 nmol TE/g DW in hydroalcoholic extracts to 22,520 ± 3304 nmol of TE/g DW in PBS extract. The antioxidant activity occurring in *C. tuberculata* and *R. pulmo* was comparably higher than in *Aurelia* sp.1 and presumably related to protein and phenol contents, although other unidentified compounds cannot be excluded. The presence of antioxidant from endosymbiotic microalgae in *C. tuberculata* [8], as well as other possible bioactive compounds in *R. pulmo*, could explain this noticeable feature. The antioxidant activity in PBS extract referring to protein content (inset in Figure 2) shows fewer but still evident differences between the Rhizostomeae jellyfish and *Aurelia*, indicating that the higher antioxidant activity could be attributable to the intrinsic protein properties of *C. tuberculata* and *R. pulmo* species.

#### 2.4.2. Antioxidant Activity of Enzymatic Hydrolyzed Peptides

The enzymatic hydrolysis resulted in a significant number of peptides able to exert considerable antioxidant activity in all pre-extracted and non-pre-extracted samples of the three jellyfish species (Figure 3). Despite the variability among samples, due to the high number of measures, several results were clear. The pepsin hydrolysis, simulating the gastric digestion, of all the treated and non-treated jellyfish samples resulted in peptides exerting antioxidant activity, which was three to five times higher in *C. tuberculata* samples than in *Aurelia* and *R. pulmo* samples (Figure 3A,B). The pre-extractions with hydroalcoholic or aqueous solutions slightly affected the antioxidant ability of peptides present in the pepsin hydrolyzed proteins, except for the *Cotylorhiza* samples, where a significantly higher antioxidant activity was detected in the non pre-treated sample. These results were more evident when the antioxidant activity was expressed as nmol TE per mg of total proteins (Figure 3B)*.* The occurrence of compounds produced by the symbiotic zooxanthellae *Symbiodinium* in *C. tuberculata* [8] may explain the higher antioxidant activity in this species as compared to the *Aurelia* and *R. pulmo*. Indeed, pre-treatment with both hydroalcoholic and aqueous solutions allowed the extraction of other soluble antioxidant compounds, which would have remained otherwise in the fraction containing the pepsin digestible peptides, contributing to the antioxidant activity of this fraction. Notably, all the pepsin-hydrolysable jellyfish proteins showed a significantly higher antioxidant activity as compared to the pepsin-hydrolysable fraction of the chicken collagen (Figure 3A,B).

A consistent amount of jellyfish peptides derived from collagenase digestion was able to exert appreciable antioxidant activity, too (Figure 3C,D). Despite the relatively homogeneous nature of the collagen, peptides derived from *C. tuberculata* and *R. pulmo* exhibited the highest antioxidant ability as compared to *Aurelia* collagen, also when data were normalized per mg of protein (Figure 3D). No significant differences among antioxidant ability of hydrolyzed collagen from pre-extracted and non-pre-extracted samples were detectable in *R. pulmo*, while *Aurelia* and *C. tuberculata* showed a higher antioxidant activity in non-pretreated samples (Figure 3C). This suggests that previous extraction and enzymatic treatments release collagen peptides with a fairly unchanged antioxidant activity (Figure 3D). However, the different antioxidant activity of hydrolyzed collagen from the three jellyfish species, particularly between the Rhizostomeae and *Aurelia*, suggests possible differences in collagen peptide composition, as also suggested from the differences in the AA composition, with particular reference to the Gly/Glu ratio (Table 2). By comparing the amount of jellyfish proteins (Table 2) and their antioxidant activity (Figure 3), the present results showed that pepsin digested peptides, while representing the smallest part of proteins, exhibit high antioxidant activity, whereas the hydrolyzed collagen, which have comparatively lower antioxidant activity, still represents the 30%–70% of the total proteins, giving overall considerable activity. In general, the *C. tuberculata* tissues seems a suitable source of pepsin digestible bioactive peptides while *R. pulmo* seems a valuable resource of collagen and collagen derived bioactive peptides, also taking into account the specimen size and yield in dry weight (Table 1).

In this study, different collagen from diverse sources (vertebrate and cnidarians) was hydrolyzed by the same enzymatic system, producing a different set of peptides with different antioxidant activity. Remarkably, the antioxidant activity exerted by hydrolyzed collagen of chicken cartilage was actually much lower than hydrolyzed collagen from all the three jellyfish (Figure 3C,D). Antioxidant compounds are known to originate by enzymatic hydrolysis of parent proteins from terrestrial and marine organisms, showing novel antihypertensive, antioxidant, antimicrobial and antiproliferative or immunomodulatory properties [46,86,87,88,89,90,91].

Many marine peptides including collagen exhibit multifunctional activities of interest for food, cosmetics and pharmaceutical industries [90,92]. In particular, studies on jellyfish proteins showed that the oral administration of collagen and collagen hydrolysate from the edible jellyfish *Rhopilema* were able to alleviate the skin photoaging in mice through antioxidant, anti-melanogenic and immunity-enhancing biochemical activities [40,67,93]. More recently, collagen hydrolysate from *Rhopilema esculentum* and the ribbon jellyfish *Chrysaora* sp. were shown to exert antioxidant and anti-hypertensive activities [94,95].

The amino acid composition of food protein hydrolysates is also known to have strong influence on their antioxidant properties. The amounts of histidine, cysteine, proline, methionine, and aromatic amino acids have been reported to significantly contribute to the antioxidant activity of food peptides. The amino acid composition of the three Mediterranean jellyfish (Table 2) investigated here shows that higher amounts of amino acids with potential antioxidant activity, including aromatic amino acids, occur in the two Rhizostomeae, *C. tuberculata* and *R. pulmo*, as compared to *Aurelia*.

### 2.5. Sodium Dodecyl Sulfate-Polyacrylamide Gel Electrophoresis (SDS-PAGE) Analysis of Hydrolyzed Peptides

**Figure 4:**
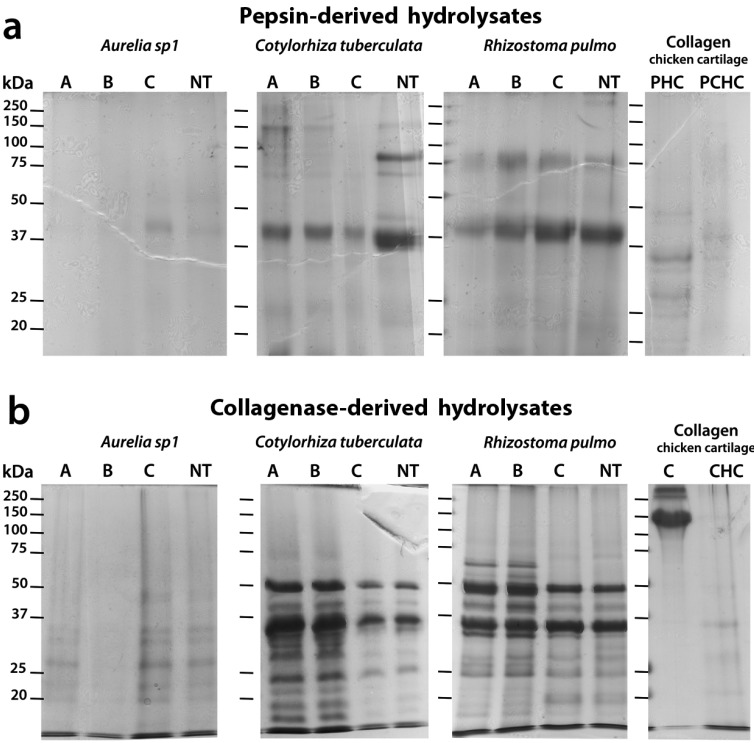
Polypeptide patterns of pepsin hyrolysates (**a**) or collagenase hyrolysates (**b**) separated by 12% reducing sodium dodecyl sulfate-polyacrylamide gel electrophoresis (SDS-PAGE). The molecular weight size marker (range of 250–15kDa), was run in parallel with samples for molecular weight estimation. Each line contained 30 μg of proteins and bands were visualized by staining gels with Coomassie Brillian Blue R-250 dye.

The SDS-PAGE electrophoretic separation of jellyfish proteins was carried out after hydrolysis with pepsin (a) and collagenase (b) on samples subjected to different solvent extractions in PBS (A), in 80% ethanol (B) or methanol (C) or on non-pre-extracted (NT) samples (Figure 4). Patterns of soluble polypeptides with a size higher than 15 kDa were compared. Polypeptide patterns derived from pepsin digestion slightly differed among jellyfish species and extraction methods (Figure 4a), with pepsin-hydrolysed peptides ranging around 30–40 kDa, 70–100 kDa and 150kDa. The patterns of soluble peptides after pepsin digestion were more complex in *C. tuberculata* and *R. pulmo* samples than in *Aurelia*, confirming the composite nature of rhizostomae species [8]. A large band of polypeptides with molecular weight around 40 kDa were common to all pepsin-hydrolysate jellyfish samples. For each jellyfish species, comparison among peptide patterns from pre-extracted and non-treated samples did not reveal major differences, indicating that the preliminary aqueous or hydroalcoholic extractions have no effect on the bulk of jellyfish proteins. The polypeptide patterns derived from collagen of chicken cartilage (C), hydrolysed by pepsin (PHC) and by pepsin followed by collagenase (PCHC), appear quite different from jellyfish samples, showing a different composition of pepsin digestible collagen in vertebrate as compared to jellyfish specimens (Figure 4a).

The patterns of peptides derived from collagenase digestion of jellyfish samples previously subjected to pepsin hydrolysis shows the bulk of peptides at low molecular weight (up to 50–70 kDa), as a result of the complete digestion (Figure 4b) with clear interspecific differences. Collagenase-treated *Aurelia* sp.1 samples showed a fewer number of peptides of molecular weight 20–50 kDa compared to *C. tuberculata* and *R. pulmo*. For the two rhizostomae jellyfish, the electrophoretic profile of hydrolysed collagen showed, two large shared bands around 35 kDa and 50 kDa and numerous species-specific polypeptides in the range of 15–50 kDa (*C. tuberculata*) and 20–70 kDa (*R. pulmo*).

By comparing hydrolysed collagen from jellyfish samples subjected to different extraction types, no or slight differences were detectable by SDS-PAGE separation, indicating that previous aqueous or hydroalcoholic extraction and pepsin digestion leave the bulk of jellyfish collagen free enough from other major proteins.

In addition, the pattern of chicken collagen proteins directly subjected to hydrolysis with the *Clostridium histolyticum* collagenase (CHC) (Figure 4b) shared a few faint bands at low molecular weight (20, 27 and 34 kDa), with the electrophoretic profile of the hydrolysed collagen from *C.* *tuberculata* and of *R. pulmo*. The different electrophoretic patterns of jellyfish hydrolysed collagen compared to vertebrate collagen indicated large structural differences among the collagen peptides.

Because of the higher antioxidant activity exerted by jellyfish hydrolysed collagen, as compared to chicken hydrolysed collagen (Figure 3A,B), the exploitation of jellyfish biomasses as functional food and/or as natural antioxidant peptide source appears to be a particularly promising strategy.

### 2.6. Lipid Content

The lipid content of the three jellyfish species showed remarkable interspecific differences (Table 4). Total lipid content was around 4 g per 100 g of DW for both *Aurelia* sp.1 and *R. pulmo*, while was three times higher (12.3 ± 0.7 g/100 g DW) in *C. tuberculata*. Lipids are incorporated to a large extent in the cell membrane systems, the higher amount of lipid content in *C.* *tuberculata* could be related to the presence of photosynthetic membranes of the endosymbiotic *Symbiodinium* [8,96].

**Table 4:** Comparison of the fatty acid composition and total lipids from jellyfish *Aurelia* sp.1, *C. tuberculata* and *R. pulmo.* Total lipids are expressed as g/100 g of dry weight ± standard deviation (SD) and fatty acid composition data are expressed as percentage of the total fatty acids ± SD.

	*Aurelia* sp1	*Cotylorhiza tuberculata*	*Rhizostoma pulmo*
Fatty acids (FA)	%		
*Saturated FA (SFA)*			
Lauric acid C_12:0_	-	-	1.3±0.5
Myristic acid C_14:0_	2.4 ± 0.6	2.9 ± 0.2	3.1 ± 0.4
Palmitic acid C_16:0_	33.0 ± 1.9	26.1 ± 0.1	33.2 ± 0.5
Margaric acid C_17:0_	1.4 ± 0.5	0.8 ± 0.1	-
Stearic acid C_18:0_	32.7 ± 1.6	24.2 ± 0.5	30.6 ± 1.8
Arachidic acid C_20:0_	-	0.8 ± 0.1	-
***Total SFA***	***69.5***	***54.8***	***68.2***
*Monounsaturated FA*			
Palmitoleic acid C_16:1_	-	1.2 ± 0.7	
Oleic acid C_18:1_ (*ω*9)	3.0 ± 0.7	12.8 ± 0.1	5.1 ± 1.8
Vaccenic acid C_18:1_ (*ω*7)	1.7 ± 0.2	1.2 ± 0.1	1.9 ± 0.5
***Total MUFA***	***4.7***	***15.2***	***7.0***
*Polyunsaturated FA (PUFA)*			
Linoleic acid C_18:2_ (*ω*6) *	1.3 ± 0.2	8.3 ± 1.6	2.5 ± 0.7
Eicosatetraenoic acid C_20:4_ (*ω*3)	-	4. 1 ± 0.3	-
Arachidonic acid C_20:4_ (*ω*6)	5.5 ± 1.1	5.3 ± 0.5	8.8 ± 0.5
Eicosapentaenoic acid C_20:5_ (*ω*3)	14.6 ± 2.2	5.1 ± 0.5	8.6 ± 1.7
Docosahexaenoic acid C_22:6_ (*ω*3)	4.4 ± 1.1	7.2 ± 0.9	4.9 ± 1.1
***Total PUFA***	***25.8***	***30.0***	***24.8***
**Σ*ω*6**	6.8	13.6	11.3
**Σ*ω*3**	19.0	16.4	13.5
***ω*6/*ω*3**	0.36	0.83	0.8
**Total Lipids** (g/100 g dry weight)	**4.1 ± 0.5**	**12.3 ± 0.7**	**4.0 ± 0.8**

SFA: saturated fatty acids; MUFA: monounsaturated fatty acids; PUFA: polyunsaturated fatty acids; Σ*ω*6: total *ω*6 fatty acids; Σ*ω*3: total *ω*3 fatty acids; *: essential fatty acid.

The fatty acid (FA) quantitative composition (as percentage values) showed similar FA profiles in the three jellyfish species (Table 4). Saturated fatty acids (SFA), accounted for two third of total FA (about 55%–70%), followed by polyunsaturated fatty acid (PUFA), representing about one third of the total FA (about 25%–30%), and a few amount of monounsaturated fatty acids (MUFA), representing about 4%–15% of the total FA. Saturated fatty acids consisted mostly of palmitic (C_16:0_) and stearic (C_18:0_) acids, followed by myristic (C_14:0_) and margaric (C_17:0_) acids. Lauric acid was detected only in *R. pulmo*, while traces of arachidic acid (C_20:0_) were detected in *C. tuberculata*. Among MUFA, oleic acid (C_18:1_) was the prevalent FA and palmitoleic acid (C_16:1_) was detected only in *C. tuberculata*. Remarkable differences were observed in the composition of PUFAs among the three jellyfish (Table 4), being mostly represented by the *ω*-3 eicosapentaenoic acid (C_20:5_) in *Aurelia* sp.1, while the *ω*-6 arachidonic (C_20:4_) and the *ω*-3 eicosapentaenoic acid (C_20:5_) were prevalent in *R. pulmo*. A peculiar PUFA composition was detected in *C. tuberculata* samples, where the essential *ω*-6 FA, linoleic acid (C_18:2_) was the major component, together with the *ω*-3 eicosatetraenoic acid (C_20:4_), docosahexaenoic acid (C_22:6_), and the eicosapentaenoic and arachidonic acids. The presence of unsaturated long chain fatty acids in this species is due to the presence of microalgal symbionts (*Symbiodinium* spp*.*), an important and significant source of essential *ω*-3 fatty acids. 

Overall, *ω*-3 PUFAs were abundant in the three jellyfish species, with the ratio of *ω*-6 to *ω*-3 resulting always in favouring *ω*-3 fatty acids (Table 4), as generally observed in fish and marine foods. The value of *ω*-6/*ω*-3 ratio was lower in *Aurelia* sp.1 (0.36) than in the two Rhizostomeae (0.8). Opposite differences in the *ω*-6/*ω*-3 ratio have been noted between fatty acid compositions of marine and freshwater fish, with marine fish containing higher levels of *ω*-3 than freshwater fish [97]. Explanations for such opposite patterns for jellyfish and fish are merely speculative. Overall, differences between marine and freshwater taxa might be related to specific requirements both to physiological adaptations to different habitats and to deep evolutionary constraints across distant phylogenetic lineages, such as jellyfish and fish.

The *ω*-3 types of FA are known to be involved in a number of biological processes including growth, development, tissue and cell homeostasis [98] and have a variety of health benefits including hypo-triglyceridemic, anti-inflammatory antihypertensive, anticancer, antioxidant, antidepressive, antiaging, and antiarthritis effects [99]. In humans, the dietary patterns are important in the pathogenesis of chronic disease, which appears to be due to proinflammatory effects of the Western diet, with particular reference to the high *ω*-6/*ω*-3 ratio [100].

## 3. Experimental Section 

### 3.1. Materials and Chemicals

Methanol, ethanol and acetic acid were purchased from Merck (Darmstadt, Germany); potassium persulfate (dipotassium peroxdisulfate), 6-hydroxy-2,5,7,8-tetramethylchroman-2-carboxylic acid (Trolox), 2,20-azinobis(3-ethylben-zothiazoline-6-sulfonic acid)diammonium salt (ABTS), gallic acid, Folin-Ciocalteu’s phenol reagent, Coomassie Brilliant Blue R-250, pepsin from porcine gastric mucosa (≥2500 U/mg), collagenase from *Clostridium histolyticum* (0.5–5.0 furylacryloyl-Leu-Gly-Pro-Ala (FALGPA) units/mg solid, ≥125 collagen digestion unit (CDU)/mg solid), collagen from chicken sternal cartilage, fatty acid methyl esters (FAME) Mix (C8–C24) and PUFA-3 were all purchased from Sigma-Aldrich (Milan, Italy). Acrylamide solution was purchased from Euroclone (Milan, Italy). All other reagents were of analytical grade.

### 3.2. Sample Collection and Preparation

Specimens of three jellyfish species (*Aurelia* sp.1, *Cotylorhiza tuberculata* and *Rhizostoma pulmo*) were collected offshore of Apulia coasts (Italy) in the 2011–2014 summers. *Aurelia* samples were collected in May 2012 in the Varano Lake (Foggia, Italy, +41°52′45.01″, +15°44′46.00″); *C. tuberculata* and *R. pulmo* samples were collected in the Southern Adriatic (Otranto, Italy) and Ionian (Castellaneta Marina and Pulsano, Italy) Seas. After the biometric measurements (weight and diameter), each specimen was frozen in liquid nitrogen and stored at −80 °C until lyophilization. Frozen jellyfish were freeze-dried for 4 days at −55 °C using a chamber pressure of 0.110 mbar in a freeze dryer (Freezone 4.5L Dry System, Labconco Co. Thermo Scientific, Milan, Italy). Lyophilized samples were weighed to annotate the dry weight and stored at −20 °C until use. 

### 3.3. Sequential Extraction and Hydrolysis

#### 3.3.1. Polar Solvent Extraction

Lyophilized samples (100 mg) of total jellyfish were subjected to extraction in hydroalcoholic (80% methanol or 80% ethanol) or aqueous solvents PBS as shown in the Scheme 1. Samples were either stirred with 16 volumes (*w*/*v*) of 80% methanol or 80% ethanol (16 h at 4 °C) or with 16 volumes of PBS (2 h at 4 °C). Samples were then centrifuged at 9000× *g* for 30 min at 4 °C and the supernatants were essayed for protein and phenol contents and antioxidant activity. The insoluble pellets were dried under a stream of nitrogen and subsequently subjected to sequential enzymatic digestions.

#### 3.3.2. Enzymatic Hydrolyses

After polar solvent extraction, the dried solid residues were subjected to proteolytic digestion with pepsin (1 mg/mL in 0.5 M acetic acid), using an enzyme /substrate ratio of 1:50 (*w*/*w*) at 4 °C for 48 h. Lyophilized samples (100 mg), not subject to extractions, were directly digested with pepsin as controls. The pepsin-digested samples were centrifuged at 9000× *g* for 30 min and the supernatants were assayed for protein and phenol contents and antioxidant activity.

Undigested pellets were washed twice with bidistilled water, centrifuged at 9000× *g* for 2 min and subjected to hydrolysis with of collagenase from *C. histolyticum* (Sigma-Aldrich) 6 mg/mL, in 50 mM 2-{[1,3-Dihydroxy-2-(hydroxymethyl)-2-propanyl]amino}ethanesulfonic acid (TES) buffer at pH 7.4 and 0.36 mM CaCl_2_, using an enzyme /substrate ratio of 1:50, at 37 °C for 5 h. As reaction control, collagen from chicken sternal cartilage (Sigma-Aldrich) was digested in the same conditions. *C. histolyticum* collagenases recognize the following peptide sequence where X is most often a neutral amino acid [101]:

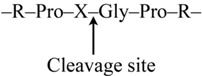



After proteolytic reaction, samples were centrifuged at 9000× *g* for 30 min, the pellets were considered as non-hydrolysable protein fractions, and supernatants were essayed for protein and phenol contents and antioxidant activity.

### 3.4. Protein Content

Total protein content was estimated by modified Bradford assay [102] using bovine serum albumin (BSA) as a standard. 

### 3.5. Amino Acidic Composition Analysis

Each sample of total dried jellyfish tissue was analysed in triplicate for the determination of free amino acids. In particular, 0.2 g of lyophilized jellyfish powder was stirred for 20 min in the following mixture: 5 mL of bidistilled water, 0.75 mL trifluoroacetic acid and 50 µL internal standard solution (5 mM dl-Norleucine in water). The extract was then centrifuged at 4 °C, 3500 rpm, for 20 min. After filtration, the extract was dried under a nitrogen stream and the residue was re-dissolved in 1 mL bidistilled water. According to the AccQ-Tag protocol (Waters, Milford, MA, USA) and after a pre-column derivatization step, each sample was analyzed on a C18 AccQ-Tag column (3.9 × 150 mm) (Waters). A gradient elution was performed according to the AccQ-Tag protocol, using phosphate buffer solution (eluent A) and acetonitrile:water 60:40 (*v*/*v*) (eluent B), the flow rate was 1 mL/min, temperature was set at 37 °C. The fluorescent detector parameters were set as follows: λ_ex_ = 250 nm, λ_em_ = 395 nm, gain = 1, EUFS (emission/energy units full scale) = 100.

### 3.6. Phenol Content

The total content of phenols was determined by a modified Folin-Ciocalteau colorimetric method. Samples (100 µL) were mixed with 500 µL of Folin-Ciocalteu’s phenol reagent and 500 µL of 7.5% sodium carbonate (Na_2_CO_3_). After 2 h of incubation at room temperature in the dark, the absorbance was spectrophotometrically measured at 760 nm. Gallic acid, ranging from 25 to 200 µg/mL, was used as a standard. The results were expressed as gallic acid equivalents (GAE) per gram of dry extract.

### 3.7. Antioxidant Activity

The total antioxidant activity was determined spectrophotometrically using the Trolox Equivalent Antioxidant Capacity (TEAC) method, as described by Longo *et al.* [103]. Ten microliters of the jellyfish samples (extracts or hydrolyzed fractions) were assayed in 1 mL of the reaction mixture and the depletion of the radical cation ABTS^+^ was measured following the decrease of absorbance at 734 nm. Comparable solutions of 80% methanol, 80% ethanol, PBS and enzymatic reaction mixtures without substrates were used as controls. A calibration curve was prepared with different concentrations of Trolox (2.5–20 μM). The antioxidant capacity of the samples was calculated as the absorbance decrease at 734 nm at 6 min as fixed time, and results were expressed as nmol of Trolox equivalents (TE) per gram of sample or per mg of contained proteins.

### 3.8. SDS-PAGE

Hydrolysed polypeptides were separated by electrophoresis through 12% polyacrylamide gels containing SDS (SDS-PAGE) as described by Leone *et al.* 2013 [8]. Precision Plus Protein Dual Color Standard (Bio-Rad, Hertfordshire, UK) was used as the molecular weight marker. Polypeptides on gels were detected by Coomassie Brilliant Blue staining (0.25% Coomassie Brilliant Blue R-250 in 10% acetic acid and 50% methanol) for 20 min followed by destaining with 10% acetic acid and 30% methanol, overnight. Molecular masses of proteins were estimated by comparing the migration of proteins of interest to the standards of known sizes.

### 3.9. Total Lipid Extraction

Total lipids were extracted using the modified method of Bligh and Dyer [104] with some modifications. Lyophilized powder (100 mg) was mixed with a total of 114 mL solvent added in this sequence: chloroform, methanol, water to achieve a final chloroform/methanol/water ratio of 1:2:0.8 (by volume). Samples were shaken for 15 s after addition of each solvent, and incubated overnight at 4 °C. After centrifugation at 6500× *g* for 10 min, the supernatant was transferred into a separating funnel, and phase separation of the biomass-solvent mixtures was achieved by adding chloroform and water to obtain a final chloroform/methanol/water ratio of 2:2:1.8 (by volume). After settling, the bottom phase was collected, evaporated in the presence of nitrogen flux and re-suspended in chloroform (1 mL).

#### Fatty Acid Profiles Determination

Fatty acid methyl esters (FAME) were obtained using boron trifluoride (BF_3_) according to [105] with some modifications. Total lipid extract (200 µL) was saponified at 90 °C for 20 min with 0.5 M KOH in methanol (3 mL). Forty-nine micrograms of the internal standard (methyl tricosanoate) were added before saponification. The fatty acids were methylated by adding 14% BF_3_ in MeOH (2 mL) and heating at 90 °C for 10 min. After cooling, hexane (1 mL) was added and vigorously stirred for 30 s before the addition of 1 mL of sodium chloride solution (0.6%). The esterified samples were placed at 4 °C for a better phase separation. After collecting the supernatant, another 1.0 mL of hexane was added and the resulting mixture was vortexed. The samples were evaporated under a stream of nitrogen, the dried samples were dissolved in 1.0 mL of hexane and analyzed by gas chromatography-mass spectrometry (GC-MS).

### 3.10. GC-MS Analysis

The analyses were performed on a GC-MS system consisted of a Shimadzu GC-17A version 3.0 coupled with MS QP5050A according to Talà *et al.* [106]. Compounds were separated on DB-5 capillary column having 30 m length, 0.25 mm ID (internal diameter) and 0.25 µm thickness. The GC parameters were as follows: the temperature of the column was 80 °C after injection then programmed at 10 °C/min to 150 °C, at 5 °C/min to 250 °C and maintained at that temperature for 15 min. Split injection was conducted with a split ratio of 50:1, the flow-rate was 1.0 mL/min, carrier gas used was 99.999% pure helium, the injector temperature was 250 °C and the column inlet pressure was 74 Kpa. The MS detection conditions were as follows: interface temperature was set 250 °C; ionization mode, EI^+^; electron energy, 70 eV; scanning method of acquisition, ranging from 30 to 450, for mass/charge (*m*/*z*) was optimized. Spectrum data were collected at 0.5 s intervals. Solvent cut time was set at 2 min and 45 min retention time sufficient for separating all the fatty acid. Compounds were identified by using online the National Institute of Standards and Technology (NIST)-library spectra and published MS data. Moreover, FAME Mix (C8–C24) and PUFA-3 (from menhaden oil) authentic standards (both from Sigma-Aldrich) were used to confirm MS data.

### 3.11. Statistical Analysis

Statistical analysis was based on a one-way ANOVA test. Tukey’s *post hoc* method was applied to establish significant differences between means (*p* < 0.05). Data are mean ± standard deviation (SD). SigmaPlot Ver 12.0 (Sistat Software, Inc., San Jose, CA, USA) was used.

## 4. Conclusions

Strategic integrative research on marine biodiversity and biotechnology is key to meeting the growing demand for healthy food in a sustainable way, calling for urgent diversification of marine food products [107]. This study demonstrates that the adult stages of three jellyfish species commonly recorded in the Mediterranean Sea in massive populations contain high amounts of collagen, anti-oxidant peptides and other bioactive molecules. These findings suggest Mediterranean jellyfish biomasses can represent a valuable source of natural compounds, not only for biomedical or pharmaceutical applications, but also as food ingredients, comparable to the most popular Asiatic jellyfish species. The biochemical and nutraceutical characterization of these gelatinous biomasses, together with adequate toxicological assays and the development of processing technologies, represent fundamental information to support future use of the vast but still unexploited resource potential of Mediterranean jellyfish.
